# Arthroscopic Treatment of Pigmented Villonodular Synovitis of the Elbow

**DOI:** 10.1155/2022/7956167

**Published:** 2022-10-13

**Authors:** Hatem B. Afana, Thomas Nau

**Affiliations:** ^1^Orthopaedic Department, King's College Hospital London, Dubai, UAE; ^2^Mohammed Bin Rashid University of Medicine and Health Sciences, Dubai, UAE

## Abstract

Pigmented villonodular synovitis (PVNS) is a benign proliferative disorder of synovium that surrounds the joints, tendon sheaths, and bursae. The elbow is rarely affected, making it difficult to establish treatment guidelines. This article relates on a case of a male patient who presented with elbow pain and decreased range of motion. Diagnosis was established with magnetic resonance imaging (MRI) and biopsy, followed by arthroscopic removal and synovectomy. The patient was pain free shortly after surgery and gained free range of motion after six weeks. At the most recent follow-up after six months, he remained clinically well. The most recent MRI did not reveal any recurrence.

## 1. Introduction

Pigmented villonodular synovitis (PVNS), today also known as tenosynovial giant cell tumor (TSGCT), is a proliferative disorder affecting the synovium of tendon sheaths, bursal membranes, and synovial joints; the most affected joint is the knee followed by the hip [1]. The traditional standard treatment of PVNS is synovectomy achieved by open or arthroscopic surgery [2], adjuvant with postoperative radiation [3, 4] to decrease the recurrence, although this is discussed controversially. Open surgical excision of PVNS is associated with the risk of infection, wound dehiscence, joint stiffness and persistent pain, and longer surgery time and hospital stay [5–7]. PVNS of the elbow is much less documented in the literature. This article presents a case of a 36-year-old male with biopsy-verified PVNS of the elbow who was treated arthroscopically and resulted in excellent outcome at 6-month follow-up.

## 2. Case Presentation

A 36-year-old male patient, middle eastern descent, presented with left elbow swelling, persistent pain, and gradual restriction of range of elbow motion for 8 months. Prior to that, he had visited a series of other facilities and had an open biopsy, done in another facility, which had already confirmed the diagnosis of PVNS. There was no history of injury or trauma, and his past medical history was uneventful.

Physical examination showed a healed scar from the open biopsy on the extensor side of his left elbow. The range of motion was restricted from 5 degrees of extension to only 90 degrees of flexion, (FE: 90.5.0), in comparison to the contralateral side with 0-degree extension to 140-degree flexion (FE: 140.0.0). Pronation and supination were free, he did not have any signs of ligament instability, and his neurovascular status was normal.

Radiographs were normal, and MRI revealed a soft tissue tumor in the anterior joint compartment with extension to the olecranon fossa and the radiocapitellar joint, with the signal intensity consistent with PVNS. There were no osteolytic lesions or osseous erosions visible on MRI.

As the suspected diagnosis of PVNS was already confirmed via biopsy, elbow arthroscopy, tumor excision, and synovectomy were indicated.

## 3. Procedure

**Figure 1:**
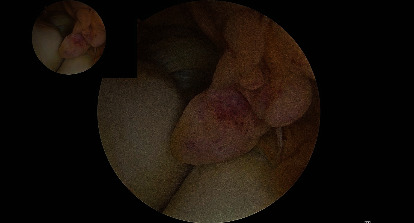
Arthroscopically view of PVNS of the elbow.

Under general anaesthesia, the patient was positioned in prone position with the left elbow supported and free for mobilisation. Tourniquet was not used. Under routine antibiotic coverage and surgical site preparation, anteromedial, anterolateral, posterolateral, and posterior portals were established. The anterior compartment was addressed first. Arthroscopic exploration showed a large soft tissue tumor, exactly as the MRI demonstrated, with the typical appearance of a nodular form of PVNS (Figure 1). The synovium showed a diffuse form of PVNS. Firstly, we removed the tumor-like nodules, followed by total synovectomy, which was performed with an arthroscopic shaver and with a radio frequency ablation device to keep bleeding to a minimum. The joint cartilage showed grade 2 changes on the distal humerus as well as on the radial head. The annular ligament was intact. After the anterior compartment was finished, the posterior compartment was addressed in a similar manner. There was some scar tissue which needed to be debrided first due to the open biopsy that had been done previously. Then, a total synovectomy was performed in the same way as for the anterior compartment. A drain was put in the anterior compartment, the wounds were closed, and the arm was immobilised in a sling for comfort.

**Figure 2:**
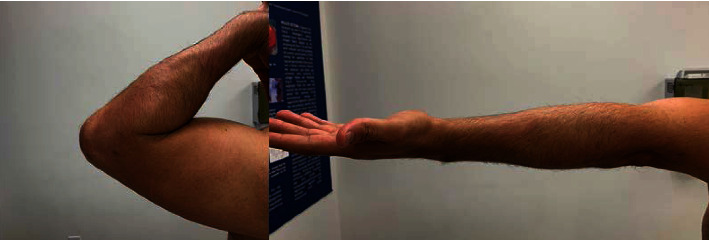
6-month postoperative range of motion.

**Figure 3:**
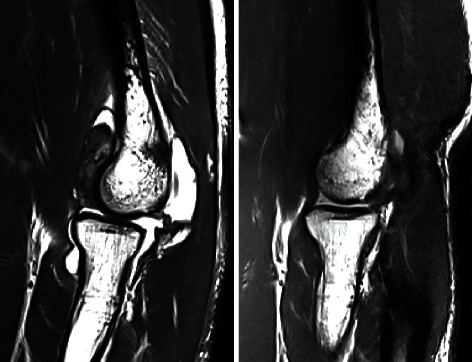
Preoperative and 6-month postoperative MRI.

The postoperative course was uneventful; rehabilitation was initiated in the first week after a few days of rest, focusing on gain of passive and active range of motion. The patient presented with a little pain but showed local swelling that persisted for two weeks. Range of motion was near normal at the end of the sixth week postoperatively (Figure 2). At the most recent follow-up six months postoperatively, the patient was pain free and had regained full range of motion, and the follow-up MRI did not reveal any recurrence (Figure 3).

## 4. Discussion

PVNS (pigmented villonodular synovitis), first described by Chassaignac in 1852 [8], is a benign, locally aggressive monoarticular proliferative disease of the synovium that rarely turns malignant and whose etiology is unknown. If left untreated, it can induce bone erosion and subsequently osteoarthritis or even amputation [9]. The synovium is a thin connective tissue layer that lines the joint, bursa, and tendon sheath and contains two types of synoviocytes: macrophage-like synovial cells (type A) which remove debris from synovial fluid and fibroblast-like synoviocytes (type B) which produce hyaluronan-rich synovial fluid that lubricates the cartilage and facilitates tendon movement. [10] PVNS is characterized by a pigmented synovium with excessive hyaluronan production [11]. The pigmentation is secondary to the deposition of hemosiderin which is an iron-containing pigment derived from partially digested ferritin, originating from an erythrocyte breakdown, when they are phagocyted by macrophage-type A synoviocytes. The outgrowing of type B synoviocyte produces large amounts of hyaluronan and synovial fluid, causing effusion and swelling and making joint movement painful [12]. PVNS can be villous, nodular, or both (villonodular) [13]. It can be localised, with limitation to synovium of the bursa or tendon sheath, or it can be diffuse, typically affecting the synovial membrane around joints [14].

Patients with PVNS most commonly report symptoms of pain, swelling, and decreased range of motion of the affected joint. On physical examination, a palpable mass with or without tenderness may be present; also, mechanical symptoms such as locking or clicking are commonly reported [15].

PVNS is difficult to be diagnosed radiologically, as scans such as X-ray, CT, and ultrasound reveal a vague irregular mass with or without bone erosion. MRI is usually done to raise the suspicion of PVNS, with characteristic presence of hemosiderin precipitate within the nodules, and is also the standard diagnostic tool to monitor an eventual recurrence at a later stage. As a result, a biopsy is the gold standard investigation needed to confirm the diagnosis [16] Histologically, PVNS is a tenosynovial giant cell tumour, characterized by proliferation of two cell types, mononuclear cells and macrophages overloaded with hemosiderin [17]. In the case reported here, an open biopsy had been in another facility.

PVNS is best treated with total or a near total synovectomy. This can be achieved open or arthroscopically and can be followed by adjuvant therapy to minimize the recurrence rate which was documented to be around 40% after synovectomy of the knee joint [8]. Radiation therapy has been shown to reduce PVNS recurrence postsurgical synovectomy significantly [3, 4] but also carries the risk of serious complications such as malignant transformation [18]. Open synovectomy has been linked to an increased risk of infection, suture dehiscence, and joint stiffness [6, 7]. As a consequence, arthroscopic tumor resection and synovectomy seem to be the preferred treatment modality for PVNS in all large joints. Recently, CSF-1 receptor modulators, emactuzumab and pexidartinib, have shown promise for treatment of PVNS. Pexidartinib was approved by the FDA for patients who are not likely to benefit from surgical intervention [18, 19].

The elbow is rarely affected by PVNS. In a recent literature review, only 27 patients with surgically treated PVNS of the elbow were reported; among them, only 4 patients were treated arthroscopically with good outcome reported for both treatment modalities [15]. The overall recurrence rate after the synovectomy of the elbow joint was around 17.4% [15]. Interestingly, nonrecurrence occurred in arthroscopically treated patients. Due to small number (4/27), the authors could not draw any strong conclusion. The literature for PVNS of the knee, where the numbers are higher, shows a similar reoccurrence rate after open and arthroscopic synovectomy [20], but with higher complication rate after open synovectomy [6, 7].

In this reported case, we used an entirely arthroscopic approach, which resulted in an excellent outcome with no recurrence after six months. However, a prospective study with a larger number of patients and longer follow-up duration is needed to fully establish the ideal treatment of PVNS of the elbow.

## 5. Conclusion

Arthroscopic synovectomy for a patient with PVNS in the elbow is a safe and effective procedure with good functional outcome.

## Data Availability

All data are available in the database of the hospital and from the author.
